# Looking Forward

**Published:** 1889-10

**Authors:** S. H. Preston


### HALL'S
### JOURNAL OF HEALTH

TRUTH DEMANDS NO SACRIFICE; ERROR CAN MAKE NONE.

VOL. 36. OCTOBER, 1889. No. 10.

### LOOKING FORWARD.

BY S. H. PRESTON.

No. 2.

Since 1750 our population has considerably more than doubled each generation. It has grown from 1,260,000, less than the present population of New York City, to 65,000,000.

At this ratio of increase the child is now born who, if he lives till his sixtieth birthday, will be the citizen of a country containing 400,000,000. And his son, then at his manhood age, at his sixtieth birthday, will belong to a great republic of 1,000,000,000 people. Figure this out for yourselves.

Taking the census of the past century as a basis of prediction, we shall, if nothing occurs to materially impede the progress of population, number one hundred years from now 1,000,000,000.

With the hunger and hard conditions of laborous life in the Old World, increased facilities of ocean travel and cheapening fares, and all the inducements to emigration of our prosperous Republic, there is no reason to reckon upon a reduction of the foreign overflow to our hospitable shores. The millions will continue to come unless Congress imposes restrictions. Which is not probable as long as pot-house politics control the country, and steamship lines the national legislature. And while Europe is exerting every effort to expatriate its poor and improvident, there is no prospect that the grade of immigrants will be improved.

Even now every line of labor is over-full, and there is more than a million who cannot get work at any wages in the United States. And we are the most prosperous people on the planet, and ours the only country that really has room for more. And this while the world's population is increasing at the rate of nearly a million a month. This is according to the statistics of Behm and Wagner, the greatest of German geographers, whose work is accepted as an authority on the subject. Its figures show that the population of the earth is now 16,778,000 more than it was nineteen months ago.

In his "Origin of Species" Darwin declares that "there is no exception to the rule that every organic being naturally increases at so high a rate that, if not destroyed, the earth would soon be covered by the progeny of a single pair. Even slow-breeding man doubles in twenty-five years, and at this rate in a few thousand years, there would literally not be standing room for his progeny."

Again he says: "Lighten any check, mitigate the destruction ever so little, and the number of the species will almost instantaneously increase to any amount."

All that breathes to-day are indebted to death, that universal destroyer, who sweeps off the generations that successive ones may have room among the ever rising reinforcements of the race. The earth is a vast cemetery, and we daily tread beneath our feet the dust of populations incomputable. According to scientific speculation the entire surface of our earth has been dug over one hundred and twenty-eight times to bury its dead. Truly.

> "All that tread
> The globe are but a handful to the tribes
> That slumber in its bosom."

John Stuart Mill likewise writes: "The power of multiplication inherent in all organic life may be regarded as infinite. There is no species of vegetable or animal which, if the earth were entirely abandoned to it, and to the things on which it feeds, would not in a small number of years, over-spread every region of the globe of which the climate was compatable with its existence." A single pair of herrings would, were their increase unchecked, in the course of time so stock the seas as to stop the paddle wheels of steamers.

So it is plain that population must be repressed in some way. The check must come either from man or from nature. Unless some wise and humane means be adopted for restricting the increase of the race, nature will step in and cut off the surplus. Her methods are merciless and cruel—such as flood and fire and famine, earthquakes, volcanic ereptions, and decimating epidemics.

During the third century a deadly disease ravaged Rome for fifteen years, carrying off 5,000 daily. In the fourteenth century, an appalling plague spread death and desolation from the Delta of the Nile to the capitals of Europe, crossed the English channel to the festive court of Edward III., and strewed its ghastly course with corpses from Cornwall to the Grampian Hills. It became known as the "Black Death," because of the horrible change it produced in the appearance of its victims. In two years it destroyed more than one-half of the inhabitants of Great Britain. As showing the relation between labor and population, it is stated that prior to the advent of this dreadful visitant a days labor would scarcely purchase a peck of wheat. Immedialely after the great mortality a day's labor would buy a bushel.

In the beginning of the sixteenth century what was called the "sweating sickness" depopulated Oxford and slew half the inhabitants of the large towns of England. In London, during the winter of 1603-4, 30,578 people perished by the plague; and in 1604-65, 68,596. In 1665, 400,000 died in Naples; and in 1792 over 800,000 in Egypt. In 1258 more than 15,000 starved to death in London, and in 1348 one-third of the whole population of France died from famine.

Not long ago nine and a half millions died of starvation in China. People enough to populate another empire perished for want of food. Districts once densely populated were left uninhabited. In 1880, Northern Brazil was wasted by a fearful famine. In one province of 900,000 inhabitants, 600,000 died of starvation. In the city of Ceara half of the population perished; 32,000 were interred in two months. Of 15,000 who sought refuge at one of the ports, 12,000 died. These others wandered off and the place was left utterly depopulated. These figures tell a startling story of starvation on the American continent, while there are other lands, like India and Ireland, perpetually on the verge of distress, and periodically decimated by wide-spread famine.

Of course there are countries that produce more than enough food for their own needs; but there are other countries, like Silesia, where semi-starvation has its permanent abode, and countries like Ireland that continually stand as beggars at the door-ways of the nations.

Wasting wars and plagues and destructive catastrophes have proved but temporary checks. The loss has been rapidly repaired, and population gone right on augmenting faster than ever. The dark old ages of brutality and blood were unpropitious for propagation; and yet most of the time there were more mouths than bread, and the fiend of famine was never far away.

Attila, Genseric, Genghis Kahn, and Tamerlane, ranged the earth like red-handed reapers in the harvest fields of life. Cities such as Pompeii, Herculaneum, and Lisbon, were swallowed up. The generations of Europe were poured into Palestine during the Crusades to perish by the hundred millions. And still population seemed to sustain scarcely a perceptible check, and ere long was redoubling faster than ever. It is a fact proved by all the past, that when population passes the limit prescribed by nature for its subsistence, nature herself will pitilessly strike the balance between the birth-rate and the death-rate.

As civilization has advanced, a new state of society has risen tending to the protection and prolongation of human life. The brain of man has been busy devising means for averting some of the most destructive checks to population. Medical and sanitary science have achieved much toward improving the conditions of life and establishing the causes of preventable disease. As people came to have better food, clothing and homes, the epidemics which once depopulated whole districts disappeared. Now there are no murderous hordes to sweep down over the steppes of Asia or swarm forth from the forests and swamp of Europe to slaughter mankind and blot out populous cities in blood—no more Attilas or Tamerlanes to ravage the race with their terrible tribes. The days for Armadas and Napoleons have departed forever. Never again will it be possible for "sweating sickness" to sweep off mankind by wholesale. The world is being disinfected, and latter day doctors have discovered agencies for driving out pestilences and epidemic diseases. Children are being better born and cared for, the lease of human life is surely being lengthened, and the race is growing more rapidly as the generations grow more enlightened and scientific. So that for the future the prospect is fair for population to double at an accelerated rate.

And this, without some great counterbalancing moral check, will but help hasten the time when mankind will have multiplied beyond the means of maintenance, and the entire earth will be as the Ireland of to-day. And the faster civilation vanquishes vice and dispels darkness, and the beneficient forces of a higher humanity drive war away, and ignorance and distress and depopulating disease, the sooner will come that calamitous over crowding.

In the history of every people on earth that have inhabited one country for several centuries are to be found certain facts related to one another as alternatives. These are migrations, devastating famines, systematic infanticide, or the wholesale slaughter of frequent wars. In China an attempt is made to repress population by a regular method of baby-killing, but this does not prevent, in an unpropitious season, the starvation of a number of people sufficient to populate one of our states.

In European countries population has been kept down by the sword of almost ceaseless conflict. At the close of the "Thirty Year's War," two and a half centuries ago, Germany was left an almost unpeopled region.

But armies are no longer swept off in a blaze of glory, and infanticide is too atrocious for these times. And but for the drain afforded by emigration, every kingdom of continental Europe ere this would have been in a condition of dire distress. All over the Old World the breadwinners are being crowded out, great numbers of them being glad to get away from their wretched homes by begging or working their passage to more prosperous lands. Some of them are starving, and all are loaded down with taxation and disabilities that reduce them to the plight of the Helots of ancient Greece.

